# Enhanced Cortical Excitability in Grapheme-Color Synesthesia and Its Modulation

**DOI:** 10.1016/j.cub.2011.10.032

**Published:** 2011-12-06

**Authors:** Devin Blair Terhune, Sarah Tai, Alan Cowey, Tudor Popescu, Roi Cohen Kadosh

**Affiliations:** 1 Department of Experimental Psychology, University of Oxford, Oxford OX1 2JD, UK; 2 Medical Sciences Division, University of Oxford, Oxford OX1 2JD, UK; 3 Centre for Functional Magnetic Resonance Imaging of the Brain, University of Oxford, Oxford OX1 2JD, UK

## Abstract

Synesthesia is an unusual condition characterized by the over-binding of two or more features and the concomitant automatic and conscious experience of atypical, ancillary images or perceptions [1–3]. Previous research suggests that synesthetes display enhanced modality-specific perceptual processing [4–7], but it remains unclear whether enhanced processing contributes to conscious awareness of color photisms. In three experiments, we investigated whether grapheme-color synesthesia is characterized by enhanced cortical excitability in primary visual cortex and the role played by this hyperexcitability in the expression of synesthesia. Using transcranial magnetic stimulation, we show that synesthetes display 3-fold lower phosphene thresholds than controls during stimulation of the primary visual cortex. We next used transcranial direct current stimulation to discriminate between two competing hypotheses of the role of hyperexcitability in the expression of synesthesia. We demonstrate that synesthesia can be selectively augmented with cathodal stimulation and attenuated with anodal stimulation of primary visual cortex. A control task revealed that the effect of the brain stimulation was specific to the experience of synesthesia. These results indicate that hyperexcitability acts as a source of noise in visual cortex that influences the availability of the neuronal signals underlying conscious awareness of synesthetic photisms.

## Highlights

► Grapheme-color synesthetes display hyperexcitability in primary visual cortex ► Synesthetes and controls do not differ in motor cortex excitability ► Synesthesia can be modulated using transcranial direct current stimulation

## Results and Discussion

Synesthesia is an unusual condition that is characterized by atypical experiences involving the over-binding of two features, such as the automatic experience of colors when perceiving or representing numbers (grapheme-color synesthesia) [1, 2]. Synesthetes exhibit functional and structural differences from nonsynesthetes in grapheme- and color-processing cortical regions [2, 8, 9]. However, the neural mechanisms underlying the conscious awareness of grapheme-color associations, which are present to a lesser extent, but nonautomatic and usually implicit, in the general population [10–13], remain poorly understood.

Recent studies have shown that synesthetes exhibit superior modality-specific perceptual processing (e.g., color discrimination) than nonsynesthetes [4–7]. Both superior visual processing [5] and conscious awareness of color photisms among synesthetes [10] might be attributed to enhanced cortical excitability in visual cortex in this population. Grapheme-color synesthesia inconsistently activates primary visual cortex (e.g., V1) and more reliably activates V4 [14], which plays a crucial role in color processing and has been afforded greater attention in theories of synesthesia [15]. However, neuroimaging techniques previously used to study synesthesia (e.g., functional magnetic resonance imaging [fMRI]) are not optimal for measuring cortical excitability in primary visual cortex and delineating its role in synesthesia because they rely on correlational, rather than causal, inference and are often based on baseline-relative measures of activity that can mask potential differences between synesthetes and nonsynesthetes [14]. In contrast, noninvasive brain stimulation techniques, such as transcranial magnetic stimulation (TMS) and transcranial direct current stimulation (TDCS), modulate regional and network neuronal activity and neurochemical concentrations [16]. We used transcranial magnetic stimulation (TMS) and transcranial direct-current stimulation (TDCS) to investigate whether grapheme-color synesthetes exhibited enhanced cortical excitability and how modulation of cortical excitability affects the experience of synesthesia.

**Figure 1:**
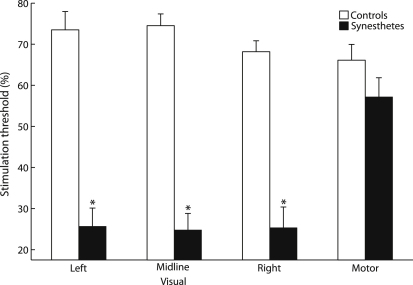
Visual and Left Primary Motor Cortex Thresholds in Controls and Grapheme-Color Synesthetes A 4 (stimulation area) × 2 (group) mixed-model analysis of variance (ANOVA) revealed that motor thresholds were higher than phosphene thresholds [F(3, 27) = 11.87, p < 0.001, η_p_^2^ = 0.57], and synesthetes displayed lower thresholds than controls [F(1, 9) = 105.15, p < 0.001, η_p_^2^ = 0.92]. These effects were qualified by a stimulation area × group interaction [F(3, 27) = 21.80, p < 0.001, η_p_^2^ = 0.71], reflecting reduced phosphene, but not motor, thresholds in synesthetes than controls. See also Table S1. Data represent mean ± 1 SEM. ^∗^p < 0.001.

We first tested the prediction that grapheme-color synesthetes would exhibit elevated cortical excitability in primary visual cortex. We recorded phosphene and motor thresholds during application of TMS, a noninvasive technique used for modulating neuronal activity [17], to primary visual and motor cortices, respectively [18]. Synesthetes exhibited approximately 300% lower phosphene thresholds than controls at left [F(1, 9) = 78.18, p < 0.001, η_p_^2^ = 0.90], mid-line [F(1, 9) = 107.06, p < 0.001, η_p_^2^ = 0.92], and right [F(1, 9) = 61.30, p < 0.001, η_p_^2^ = 0.87] visual cortex, whereas the two groups did not differ in motor thresholds (F < 1.25, p > 0.30) (Figure 1). These results demonstrate that grapheme-color synesthetes display enhanced cortical excitability that is specific to primary visual cortex.

Regional hyperexcitability might exercise a functional role in the expression of synesthesia by governing the extent to which concurrent cortical events breach conscious awareness [19] (functional hypothesis). Specifically, hyperexcitability in primary visual cortex may directly enable the conscious experience of grapheme-color associations that are otherwise unavailable to awareness. Alternatively, hyperexcitability may no longer be functionally relevant at later developmental stages due to specialization of visual cortex [20]. However, hyperexcitability would still produce activation that would act as a source of competition with other regions supporting synesthesia and thereby would be expected to reduce the signal-to-noise (SNR) ratio underlying synesthesia (SNR hypothesis).

To discriminate between these two competing hypotheses, we applied TDCS to the primary visual cortices of grapheme-color synesthetes during a task that evoked synesthetic photisms (digit-color priming [21]) and a nonsynesthetic control task (the numerical Stroop task [22]). TDCS involves the application of a constant, weak current to reduce (cathodal stimulation) or enhance (anodal stimulation) cortical excitability in the region beneath the electrode and to modulate neurotransmitters that are involved in inhibition and excitation of the central nervous system, such as γ-aminobutyric acid (GABA) and glutamate, respectively [16].

**Figure 2:**
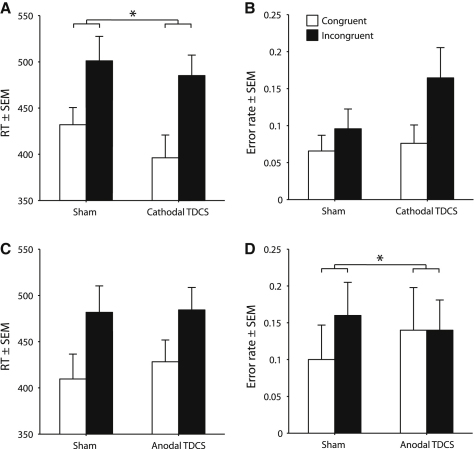
Digit-Color Priming Performance as a Function of Stimulation Applied to Primary Visual Cortex (A) Cathodal stimulation increased response time (RT) interference effects relative to sham stimulation. (B) Cathodal and sham stimulation did not differentially affect error rate interference effects. (C) Anodal and sham stimulation did not differentially affect RT interference effects. (D) Anodal stimulation reduced error rate interference effects relative to sham stimulation. See also Table S2. Data represent mean ± 1 SEM. ^∗^p < 0.05.

The functional hypothesis states that hyperexcitability directly facilitates the conscious experience of synesthetic photisms and thus predicts attenuation of synesthesia during cathodal TDCS and augmentation during anodal TDCS. In contrast, the SNR hypothesis proposes that hyperexcitability produces excess noise in visual cortex and thereby reduces conscious awareness of photisms. This hypothesis thus predicts the converse pattern: cathodal TDCS should enhance synesthesia whereas anodal TDCS should reduce it. The results of both TDCS experiments are consistent with the SNR hypothesis. In comparison with sham stimulation, cathodal TDCS applied to primary visual cortex enhanced synesthesia, as reflected in a greater behavioral interference effect, in the synesthetic digit-color priming task [condition × congruency interaction on response times (RTs), F(1, 4) = 8.53, p = 0.043, η_p_^2^ = 0.68 (Figure 2A), but not on error rates, F < 4.5, p > 0.10 (Figure 2B)]. In contrast, anodal stimulation decreased the interference effect relative to sham stimulation [condition × congruency interaction on error rates, F(1,4) = 19.28, p = 0.012, η_p_^2^ = 0.83 (Figure 2D), but not on RTs, F < 1, p > 0.40, (Figure 2C)].

Whereas the interference difference between real and sham stimulation observed in the cathodal TDCS experiment was present in the RT data, the corresponding effect in the anodal TDCS experiment occurred in the error rate data. This shift is consistent with previous findings showing that a repetition of an experiment by the same participants, even after a few months, may lead to a transition from latency effects to accuracy effects [23]. This might be due to a shift in strategies to perform the second experiment faster [24].

To control for the influence of differential speed-accuracy tradeoff effects across the cathodal and anodal TDCS experiments, we contrasted accuracy-corrected RTs (efficiency: RT / [1 – error rate]) [25] across stimulation conditions. This analysis confirmed that in the cathodal TDCS experiment the magnitude of the interference effect (incongruent – congruent) was greater in the cathodal (M ± SEM: 158 ms ± 62) than the sham (94 ms ± 82) condition, paired-samples t(4) = 2.36, p = 0.039, one-tailed, d = 0.62. Similarly, in the anodal TDCS experiment, we confirmed that the magnitude of the interference effect (incongruent – congruent) was smaller in the anodal (58 ms ± 63) than the sham (114 ms ± 72) condition, paired-samples t(4) = 2.89, p = 0.022, one-tailed, d = 0.41. At the phenomenological level, synesthetes spontaneously reported enhanced and diminished synesthetic experiences during cathodal and anodal simulation, respectively. These results confirm the RT and error rate analyses and support the SNR hypothesis and indicate that the experience of synesthesia, as reflected in behavioral responses, is inversely related to the magnitude of cortical excitability as modulated by TDCS.

**Figure 3:**
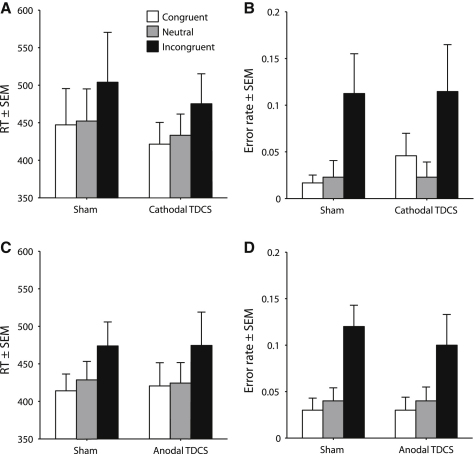
Numerical Stroop Performance as a Function of Stimulation Applied to Primary Visual Cortex (A and B) Cathodal and sham stimulation do not differentially affect numerical Stroop performance in RTs or error rates. (C and D) Anodal and sham stimulation do not differentially affect numerical Stroop performance in RTs or error rates. See also Table S3. Data represent mean ± 1 SEM.

The numerical Stroop task [22] was used as a control task to ensure that any modulatory effect of TDCS was restricted to synesthesia. Participants consistently exhibited numerical Stroop interference effects [22] across stimulation conditions (see Figure 3 and Supplemental Results), but performance did not differ across cathodal and sham conditions (condition effects and condition × congruency interactions, Fs < 1, ps > 0.4; Figures 3A and 3B) or anodal and sham conditions (condition effects and condition × congruency interactions, Fs < 0.5, ps > 0.7; Figures 3C and 3D). These results indicate that the effects of the TDCS were specific to synesthesia.

Using noninvasive brain stimulation techniques, we show that grapheme-color synesthesia is associated with hyperexcitability in primary visual cortex and that alteration of cortical excitability modulates the experience of synesthesia. These results provide a novel perspective on the neural basis of synesthesia. In accordance with Hebb's rule [26], at an early developmental stage, genetically-based [27, 28] enhanced cortical excitability among synesthetes might contribute to the establishment of atypical binding of grapheme-color associations during environmental exposure [29, 30], resulting in conscious awareness of these associations and concomitant greater gray and white matter density in grapheme- and color- processing regions [3, 8, 9]. Hyperexcitability may also give rise to enhanced domain-specific perceptual processing in grapheme-color synesthesia [4–6] as well as increased gray matter density in V1 in synesthesias with color concurrents [31–33].

However, at later developmental stages, visual cortical hyperexcitability appears no longer to exercise a direct functional role in synesthetic experience, plausibly because of maturation and cortical specialization of the visual system [20]. Rather, our results indicate that hyperexcitability assumes a different role and acts as a source of noise in visual cortex, competing with other regions supporting synesthesia, such as V4 [9, 34, 35], and thereby modulates the signal-to-noise ratio underlying the experience of synesthetic color photisms (see also Supplemental Discussion). Increasing or reducing baseline cortical excitability thus augments or attenuates synesthesia, by diminishing or enhancing, respectively, the magnitude of activation of concurrent neuronal patterns in primary visual cortex that compete with regions supporting synesthesia. This interpretation is consistent with TDCS experiments in nonsynesthetes showing enhancement of performance through reduction of neuronal noise [36, 37] and may explain inconsistent V1 activation during the experience of synesthesia [14]. These results, although limited by the number of synesthetes, could guide research examining modality-specific cortical excitability in other forms of synesthesia and investigations of how neurochemicals, such as GABA or glutamate, affect the occurrence and development of synesthesia, thus implicating models of conscious awareness in the visual domain.

## Experimental Procedures

### Participants

Six grapheme-color synesthetes, who did not have sound-color synesthesia (five women, *M*_Age_ = 21, SD = 2), and six controls (five women, *M*_Age_ = 21, SD = 1), all right-handed, participated in the TMS experiment. Six synesthetes (all women, *M*_Age_ = 21, SD = 2) volunteered for the cathodal TDCS experiment, five of whom participated in the TMS experiments. Five of these synesthetes subsequently participated in the anodal TDCS experiment (for further details, see Supplemental Experimental Procedures).

### TMS

TMS was applied to left, midline, and right primary visual and left motor cortices with closed eyes [18]. For visual cortex sites, the minimum intensity that reliably elicited phosphenes (report of phosphenes in at least five out of ten trials) was recorded as the phosphene threshold. The lowest intensity at which a motor twitch was reliably observed in the right hand (muscle movement observed in at least five out of ten trials) was recorded as the motor threshold (see Supplemental Experimental Procedures).

### TDCS

TDCS was delivered through a pair of 5 × 5 cm electrodes in saline-soaked sponges. In the cathodal experiment, the cathode was attached 2 cm above the inion and the anode was attached to the supraorbital area [38, 39]. The latter site has been used extensively in TDCS experiments [39] and has been shown to not affect cognitive functions subserved by the prefrontal cortex (see Supplemental Discussion); this is corroborated by the results with the control task. Participants completed the digit-color priming and control tasks under cathodal and sham (counterbalanced) stimulation conditions. This experimental procedure was strictly replicated in the anodal TDCS experiment but with a reverse electrode montage.

### Tasks

Participants completed a digit-color priming task to measure the magnitude of synesthetic interference [21]. Participants were presented with one of four achromatic digit primes that evoked color photisms followed by one of four color targets. The digit prime and color target were congruent on 50% of the trials. Participants identified the color of the target with motor responses.

Participants also completed the numerical Stroop task [22] as a control task. In this task, two Arabic digits were presented simultaneously on the horizontal axis in different physical sizes. Participants were instructed to ignore the digit's numerical values and identify which digit was physically larger. Stimuli were presented in congruent (physical and numerical size agreement), neutral (numerical size agreement), or incongruent (physical and numerical size in disagreement) conditions (each 33% of trials).

## Supplemental Information

Document S1. Supplemental Results, Supplemental Experimental Procedures, and Supplemental Discussion

## References

[1] Rich A.N., Mattingley J.B. (2002). Anomalous perception in synaesthesia: A cognitive neuroscience perspective. Nature reviews.

[2] Hubbard E.M., Ramachandran V.S. (2005). Neurocognitive mechanisms of synesthesia. Neuron.

[3] Bargary G., Mitchell K.J. (2008). Synaesthesia and cortical connectivity. Trends Neurosci..

[4] Barnett K.J., Foxe J.J., Molholm S., Kelly S.P., Shalgi S., Mitchell K.J., Newell F.N. (2008). Differences in early sensory-perceptual processing in synesthesia: a visual evoked potential study. Neuroimage.

[5] Banissy M.J., Walsh V., Ward J. (2009). Enhanced sensory perception in synaesthesia. Exp. Brain Res..

[6] Yaro C., Ward J. (2007). Searching for Shereshevskii: what is superior about the memory of synaesthetes?. Q J Exp Psychol (Hove).

[7] Cohen Kadosh R., Gertner L., Terhune D.B. (2011). Exceptional abilities in the spatial representation of numbers and time: Insights from synesthesia. Neuroscientist.

[8] Rouw R., Scholte H.S. (2007). Increased structural connectivity in grapheme-color synesthesia. Nat. Neurosci..

[9] Weiss P.H., Fink G.R. (2009). Grapheme-colour synaesthetes show increased grey matter volumes of parietal and fusiform cortex. Brain.

[10] Cohen Kadosh R., Henik A. (2007). Can synaesthesia research inform cognitive science?. Trends Cogn. Sci. (Regul. Ed.).

[11] Simner J., Ward J., Lanz M., Jansari A., Noonan K., Glover L., Oakley D.A. (2005). Non-random associations of graphemes to colours in synaesthetic and non-synaesthetic populations. Cogn. Neuropsychol..

[12] Rich A.N., Bradshaw J.L., Mattingley J.B. (2005). A systematic, large-scale study of synaesthesia: implications for the role of early experience in lexical-colour associations. Cognition.

[13] Ward J., Huckstep B., Tsakanikos E. (2006). Sound-colour synaesthesia: to what extent does it use cross-modal mechanisms common to us all?. Cortex.

[14] Rouw R., Scholte H.S., Colizoli O. (2011). Brain areas involved in synaesthesia: a review. J. Neuropsychol..

[15] Hubbard E.M. (2007). A real red-letter day. Nat. Neurosci..

[16] Stagg C.J., Nitsche M.A. (2011). Physiological basis of transcranial direct current stimulation. Neuroscientist.

[17] Ziemann U. (2011). Transcranial magnetic stimulation at the interface with other techniques: a powerful tool for studying the human cortex. Neuroscientist.

[18] Stewart L.M., Walsh V., Rothwell J.C. (2001). Motor and phosphene thresholds: a transcranial magnetic stimulation correlation study. Neuropsychologia.

[19] Dehaene S., Changeux J.P., Naccache L., Sackur J., Sergent C. (2006). Conscious, preconscious, and subliminal processing: a testable taxonomy. Trends Cogn. Sci. (Regul. Ed.).

[20] Johnson M.H., Grossmann T., Cohen Kadosh K. (2009). Mapping functional brain development: Building a social brain through interactive specialization. Dev. Psychol..

[21] Gebuis T., Nijboer T.C., Van der Smagt M.J. (2009). Multiple dimensions in bi-directional synesthesia. Eur. J. Neurosci..

[22] Henik A., Tzelgov J. (1982). Is three greater than five: the relation between physical and semantic size in comparison tasks. Mem. Cognit..

[23] Cohen Kadosh R., Muggleton N., Silvanto J., Walsh V. (2010). Double dissociation of format-dependent and number-specific neurons in human parietal cortex. Cereb. Cortex.

[24] Pachella R., Kantowitz B.H. (1974). The interpretation of reaction time in information processing research. Human Information Processing: Tutorials in Performance and Cognition.

[25] Mevorach C., Humphreys G.W., Shalev L. (2006). Opposite biases in salience-based selection for the left and right posterior parietal cortex. Nat. Neurosci..

[26] Hebb D.O. (1949). The Organization of Behavior.

[27] Asher J.E., Lamb J.A., Brocklebank D., Cazier J.B., Maestrini E., Addis L., Sen M., Baron-Cohen S., Monaco A.P. (2009). A whole-genome scan and fine-mapping linkage study of auditory-visual synesthesia reveals evidence of linkage to chromosomes 2q24, 5q33, 6p12, and 12p12. Am. J. Hum. Genet..

[28] Tomson S.N., Avidan N., Lee K., Sarma A.K., Tushe R., Milewicz D.M., Bray M., Leal S.M., Eagleman D.M. (2011). The genetics of colored sequence synesthesia: suggestive evidence of linkage to 16q and genetic heterogeneity for the condition. Behav. Brain Res..

[29] Witthoft N., Winawer J. (2006). Synesthetic colors determined by having colored refrigerator magnets in childhood. Cortex.

[30] Brang D., Rouw R., Ramachandran V.S., Coulson S. (2011). Similarly shaped letters evoke similar colors in grapheme-color synesthesia. Neuropsychologia.

[31] Hänggi J., Beeli G., Oechslin M.S., Jäncke L. (2008). The multiple synaesthete E.S.: neuroanatomical basis of interval-taste and tone-colour synaesthesia. Neuroimage.

[32] Jäncke L., Beeli G., Eulig C., Hänggi J. (2009). The neuroanatomy of grapheme-color synesthesia. Eur. J. Neurosci..

[33] Rouw R., Scholte H.S. (2010). Neural basis of individual differences in synesthetic experiences. J. Neurosci..

[34] Brang D., Hubbard E.M., Coulson S., Huang M., Ramachandran V.S. (2010). Magnetoencephalography reveals early activation of V4 in grapheme-color synesthesia. Neuroimage.

[35] Hubbard E.M., Arman A.C., Ramachandran V.S., Boynton G.M. (2005). Individual differences among grapheme-color synesthetes: brain-behavior correlations. Neuron.

[36] Antal A., Nitsche M.A., Kruse W., Kincses T.Z., Hoffmann K.P., Paulus W. (2004). Direct current stimulation over V5 enhances visuomotor coordination by improving motion perception in humans. J. Cogn. Neurosci..

[37] Dockery C.A., Hueckel-Weng R., Birbaumer N., Plewnia C. (2009). Enhancement of planning ability by transcranial direct current stimulation. J. Neurosci..

[38] Utz K.S., Dimova V., Oppenländer K., Kerkhoff G. (2010). Electrified minds: transcranial direct current stimulation (tDCS) and galvanic vestibular stimulation (GVS) as methods of non-invasive brain stimulation in neuropsychology—a review of current data and future implications. Neuropsychologia.

[39] Nitsche M.A., Cohen L.G., Wassermann E.M., Priori A., Lang N., Antal A., Paulus W., Hummel F., Boggio P.S., Fregni F., Pascual-Leone A. (2008). Transcranial direct current stimulation: State of the art 2008. Brain Stimulat..

