# Supplementary material for: Enhanced Cortical Excitability in Grapheme-Color Synesthesia and Its Modulation
Source: Curr Biol. 2011 Dec 6;21(23):2006–9. doi: 10.1016/j.cub.2011.10.032 (PMC3242051; doi:10.1016/j.cub.2011.10.032)
Supplement: Document S1. Supplemental Results, Supplemental Experimental Procedures, and Supplemental Discussion [file mmc1.pdf]

Current Biology, Volume *21*

Supplemental Information

Enhanced Cortical Excitability in Grapheme-Color Synesthesia and Its Modulation

Devin Blair Terhune, Sarah Tai, Alan Cowey, Tudor Popescu, and Roi Cohen Kadosh

Supplemental Inventory

1. Supplemental Tables

Table S1

Table S2

Table S3

2. Supplemental Results

3. Supplemental Discussion

4. Supplemental Experimental Procedures

5. Supplemental References

Table S1. Descriptive Statistics [M and (SEM)] for Phosphene (Primary Visual Cortex) and Motor Thresholds in Controls and Synesthetes

Region		Controls	Synaesthetes
**Visual**			
	**Left**	.74 (.04)	.22 (.03)
	**Midline**	.75 (.03)	.25 (.04)
	**Right**	.68 (.03)	.23 (.05)
**Motor**		.66 (.04)	.61 (.04)

Table S2. Descriptive Statistics [M and (SEM)] for the Conditions in the Digit-Color Priming Task in the Cathodal and Anodal TDCS Experiments

	**Cathodal TDCS**			
	**Sham**		**Cathodal**	
	Congruent	Incongruent	Congruent	Incongruent
**Error rates**	.07 (.02)	.10 (.03)	.08 (.02)	.16 (.04)
**Response times**	432 (18)	501 (26)	396 (25)	485 (22)
	**Anodal TDCS**			
	**Sham**		**Anodal**	
	Congruent	Incongruent	Congruent	Incongruent
**Error rates**	.10 (.05)	.16 (.04)	.14 (.06)	.14 (.04)
**Response times**	410 (27)	482 (29)	428 (24)	484 (24)

Table S3. Descriptive Statistics [M and (SEM)] for the Conditions in the Numerical Stroop Task in the Cathodal and Anodal TDCS Experiments

	**Cathodal TDCS**					
	**Sham**			**Cathodal**		
	Congruent	Neutral	Incongruent	Congruent	Neutral	Incongruent
**Error rates**	.02 (.01)	.02 (.02)	.11 (.04)	.05 (.02)	.02 (.02)	.11 (.05)
**Response times**	447 (48)	452 (43)	504 (66)	422 (29)	433 (28)	475 (40)

	**Anodal TDCS**					
	**Sham**			**Anodal**		
	Congruent	Neutral	Incongruent	Congruent	Neutral	Incongruent
**Error rates**	.03 (.01)	.04 (.01)	.12 (.02)	.03 (.01)	.04 (.01)	.10 (.03)
**Response times**	414 (22)	429 (24)	474 (32)	421 (31)	424 (27)	475 (44)

Supplemental Results

TMS

Descriptive statistics for the phosphene and motor thresholds are presented in Table S1.

Cathodal TDCS

We excluded one participant's data because of excessive error rates (> .25) in all conditions of the digit-colour priming task, in line with the protocol of Gebuis et al. [1], resulting in a sample of five synaesthetes. Descriptive statistics for the digit-colour priming task are presented in Table S2.

Digit-colour priming data were analyzed with 2 (TDCS: sham vs. cathodal) × 2 (Congruency: congruent vs. incongruent) repeated-measures ANOVAs. For RTs, there were neither effects of Condition, $F < 3.25$, nor Congruency, $F(1, 4) = 5.40$, $p = .081$. On error rates, there were suggestive effects of Condition, $F(1, 4) = 6.72$, $p = .061$, $\eta_p^2 = .63$, and Congruency, $F(1, 4) = 5.88$, $p = .072$, $\eta_p^2 = .60$.

Descriptive statistics for the numerical Stroop task are presented in Table S3. In line with previous findings, we observed numerical Stroop effects (poorer performance on incongruent trials) but they did not vary between cathodal and sham stimulation (Figure 3A,B). Only the main effect of Congruency was significant for both RTs, $F(2, 8) = 8.74$, $p = .010$, $\eta_p^2 = .69$, and error rates, $F(2, 8) = 5.69$, $p = .029$, $\eta_p^2 = .59$.

Anodal TDCS

Descriptive statistics for the digit-colour priming are presented in Table S2. In the digit-colour priming task, there were no main effects of Congruency or Group on RTs, *F*s < 2.5, or error rates, *F*s < 1.25.

Descriptive statistics for the numerical Stroop task are presented in Table S3. Anodal and sham stimulation did not differentially affect performance on the numerical Stroop task (Figure 3C,D). As in the cathodal TDCS experiment, only the main effects of Congruency on RTs, $F(2, 8) = 13.28$, $p = .003$, $\eta_p^2 = .77$, and error rates, $F(2, 8) = 9.88$, $p = .007$, $\eta_p^2 = .71$ were significant.

Supplemental Discussion

One potential confound in our study is the use of the supraorbital area as the reference electrode site in the TDCS experiments. Whilst our design cannot dismiss the possibility that this area contributes to the observed effects, our results indicate that it is unlikely. The idea that the use of the supraorbital area as a reference site represents a potential confound stems from the fact that this area is adjacent to the prefrontal cortex, which has previously been found to be activated during synaesthesia (for a review, see [2]). Stimulation of the supraorbital area, and not V1, may thus have produced the observed changes in the digit-colour priming task. However, frontal activations during synaesthesia probably reflect cognitive control processes recruited to selectively adjust attention in the wake of response conflict produced by synaesthesia [3]. That is, any activation of prefrontal cortex during synaesthesia is likely a byproduct related to cognitive control and not a direct neural correlate of synaesthesia. Taking this into account, cathodal stimulation of prefrontal cortex would be expected to weaken cognitive control, producing larger interference effects on the digit-colour priming task, whereas anodal stimulation would be expected to enhance control, resulting in a smaller interference effect, as observed in the TDCS experiments. However, these predictions also hold true for the numerical Stroop task, which recruits similar control processes [4-6]. Crucially, there were no TDCS effects on the numerical Stroop in either the cathodal or anodal experiments. This fits with other studies that have used TDCS to modulate prefrontal cortex excitability and used the supraorbital area as the reference site [e.g., 7] and the use of this site in TDCS experiments more broadly [8]. Accordingly, it is highly unlikely that the observed modulation of synaesthesia resulted from the use of the supraorbital area as the reference site.

An open question is whether cathodal TDCS reduces hyperexcitability of primary visual cortex in synaesthetes to the level found in non-synaesthete controls in absolute terms (as in our TMS experiment) or whether cathodal TDCS induces only a relative change with regard to synaesthetes' baseline state of hyperexcitability. Although we did not aim to answer this question, we suggest that the decrease in excitability in primary visual cortex during cathodal TDCS represents a change relative to synaesthetes' baseline state of excitability and is not sufficient to produce a level of excitability similar to that observed in controls. Previous research has shown that cathodal TDCS reduces cortical excitability in primary visual cortex (as measured by increases in phosphene threshold) by less than 20% [9]. Our TMS results suggest that cortical excitability would have to be decreased by approximately 300% to match the level observed in our sample of controls. Cumulatively, these findings suggest that the reduction in cortical excitability in synaesthetes produced by cathodal TDCS is a small change that impacts awareness of synaesthetic concurrents but does not produce a state of cortical excitability similar to controls.

Supplemental Experimental Procedures

Participants

All participants were unaware of the purpose of the experiment and gave informed written consent in accordance with ethical approval from a local ethics committee. The participants had normal or corrected-to-normal visiuon, no metallic implants or electrical devices, no history of substance abuse, migraines, or neurological or psychiatric disorders, and were not taking any medication. Test-retest reliability of synaesthetes' digit-colour pairs, recorded on two separate occasions separated by four weeks, was high with all consistency scores < 0.1 (scores < 1 are interpreted as reflecting genuine synaesthesia) [10].

TMS

Participants wore a tight lycra cap and received TMS from a Magstim TM model (Magstim, UK) via a 70 mm figure-of-eight coil in a dark room. For the visual cortex stimulation, the TMS coil was first placed with the handle in the horizontal position and the centre of the confluence of the two coils on the midline of the skull 2cm above the inion, corresponding approximately to the representation of the fovea-macula in V1. Three-pulse trains were delivered 100ms apart first at 50% intensity of the stimulator threshold (maximum stimulator output was 2 Tesla) and then in 5% shifts in intensity from the maximum stimulator output separated by at least 5s. This procedure was repeated then with left and right visual cortex at distances of 2cm to the left and right of the central stimulation area, corresponding respectively to the representation of the right and left hemi-macular in V1 and V2. TMS sounds may produce colour photisms in individuals with sound-colour synaesthesia. Importantly, none of the synaesthetes in this study reported having this form of synaesthesia. Moreover, no participants reported phosphenes when the same pulse train was applied to the vertex at 50% intensity (well above the threshold for the synaesthetes), thereby confirming that none of the participants had sound-colour synaesthesia, and eliminating this as a confound.

Left motor cortex was stimulated with the same pulse train. The stimulated region consisted of a triangle with points at the vertex, 5cm lateral and left of the vertex, and 2cm rostral of the lateral point. The coil was placed tangentially to the scalp, with the handle pointing 45° postero-laterally, whilst participants pressed together the index finger and thumb of the right hand. The stimulation started at 50% intensity and was gradually adjusted so that only the dorsal interosseus muscle of the right hand moved.

Threshold data for one synaesthete from central visual cortex was not recorded because of a technical error; this participant's data were omitted from the analyses.

TDCS

TDCS was delivered by a battery-driven, constant-current stimulator (Magstim, UK). For both the cathodal and anodal TDCS experiments, the current intensity was first raised in a ramp-like fashion (15s fade-in) until 1 mA was reached. In the TDCS condition, the current was applied for 30 minutes, whereas in the sham condition, the current decayed (15s fade-out) immediately after the fade-in. Both forms of stimulation produce a brief tingling sensation and nothing thereafter and thus are indistinguishable [8, 11].

Tasks

In the digit-priming task, digit primes and colour targets (width = 1.6°, height = 2.1°) were centrally presented on a gray background. Each trial began with a fixation cross (300ms), followed by the digit prime (500ms), an inter-stimulus interval (500ms), the colour target (500ms), and a jittered inter-trial interval (400-600ms). Participants had to identify the colour of the target using the keyboard keys *V*, *B*, *N*, and *M*, using the middle and index finger of both hands. Participants completed 20 practice trials and four experimental blocks of 96 trials. Previous experiments [1] have shown that, as expected, synaesthetes, but not non-synaesthete controls, exhibit interference effects in this task.

In the numerical Stroop task two Arabic digits (range: 1-4, 6-9) were presented simultaneously in white against a black background on the horizontal axis. One of the digits was always physically larger but the larger digit (width = 0.6°, height = 1.4°) could be numerically smaller or larger than the physically smaller digit (width = 0.5°, height = 1.2°). Alternatively, both digits could have the same numerical value (neutral trials). Participants had to indicate which digit was physically larger by pressing the *Q* key (if the left digit was larger) or the *P* key (if the right digit was larger), whilst ignoring their numerical values. Each trial began with a fixation cross (300ms), followed by a blank screen (300ms), the stimulus (500ms), and an inter-stimulus interval (200ms). Stimuli were presented in congruent (physical and numerical size in agreement, 2 4), incongruent (physical and numerical size in disagreement, 4 2), and neutral (numerical size was equivalent in the two stimuli, 2 2) conditions, with 96 trials in each condition.

Both tasks were presented on a 15” monitor using E-Prime v. 2.0 (Psychological Software Tools, Pittsburgh, PA) and were completed by participants immediately after the onset of the TDCS, as has been done in numerous online TDCS studies [10]. The cathodal experiment preceded the anodal experiment by three months. The same experimenter conducted all experiments.

References

1. Gebuis, T., Nijboer, T.C., and Van der Smagt, M.J. (2009). Multiple dimensions in bi-directional synesthesia. Eur. J. Neurosci. *29*, 1703-1710.
2. Rouw, R., Scholte, H.S., and Colizoli, O. (2011). Brain areas involved in synaesthesia: A review. J Neuropsychol *5*, 214-242.
3. Botvinick, M.M., Braver, T.S., Barch, D.M., Carter, C.S., and Cohen, J.D. (2001). Conflict monitoring and cognitive control. Psychol. Rev. *108*, 624-652.
4. Kaufmann, L., Koppelstaetter, F., Delazer, M., Siedentopf, C., Rhomberg, P., Golaszewski, S., Felber, S., and Ischebeck, A. (2005). Neural correlates of distance and congruity effects in a numerical Stroop task: An event-related fMRI study. Neuroimage *25*, 888-898.
5. Pinel, P., Piazza, M., Le Bihan, D., and Dehaene, S. (2004). Distributed and overlapping cerebral representations of number, size, and luminance during comparative judgments. Neuron *41*, 983-993.
6. Cohen Kadosh, R., Cohen Kadosh, K., Linden, D.E., Gevers, W., Berger, A., and Henik, A. (2007). The brain locus of interaction between number and size: A combined functional magnetic resonance imaging and event-related potential study. J. Cogn. Neurosci. *19*, 957-970.
7. Cerruti, C., and Schlaug, G. (2009). Anodal transcranial direct current stimulation of the prefrontal cortex enhances complex verbal associative thought. J. Cogn. Neurosci. *21*, 1980-1987.
8. Nitsche, M.A., Cohen, L.G., Wassermann, E.M., Priori, A., Lang, N., Antal, A., Paulus, W., Hummel, F., Boggio, P.S., Fregni, F., et al. (2008). Transcranial direct current stimulation: State of the art 2008. Brain stimulation *1*, 206-223.
9. Antal, A., Kincses, T.Z., Nitsche, M.A., and Paulus, W. (2003). Manipulation of phosphene thresholds by transcranial direct current stimulation in man. Exp. Brain Res. *150*, 375-378.
10. Eagleman, D.M., Kagan, A.D., Nelson, S.S., Sagaram, D., and Sarma, A.K. (2007). A standardized test battery for the study of synesthesia. J. Neurosci. Methods *159*, 139-145.
11. Utz, K.S., Dimova, V., Oppenlander, K., and Kerkhoff, G. (2010). Electrified minds: Transcranial direct current stimulation (tDCS) and galvanic vestibular stimulation (GVS) as methods of non-invasive brain stimulation in neuropsychology--a review of current data and future implications. Neuropsychologia *48*, 2789-2810.
